# The Guinea worm eradication effort: lessons for the future.

**DOI:** 10.3201/eid0403.980319

**Published:** 1998

**Authors:** D R Hopkins

**Affiliations:** The Carter Center, Atlanta, Georgia, USA.


### The Guinea Worm Eradication Effort: Lessons for the Future

**Donald R. Hopkins**
The Carter Center, Atlanta, Georgia, USA

The dracunculiasis (Guinea worm) eradication campaign has specific implications for efforts to control other emerging infectious diseases. Guinea worm, a painful disfiguring disease, affects primarily adults, who often become ill in very large numbers (usually 30% or more of a village's population) during the planting or harvest season. The disease used to be transmitted in parts of Asia and in Africa in open standing stagnant water. The intermediate host of the parasite, the copepod, contains the larva of the worm in such open drinking water; these organisms are barely visible in a glass of drinking water held up to the light. Thirteen years ago, the disease was still endemic in parts of the Indian subcontinent, a small part of Pakistan and India, Yemen, and the band of countries across Africa from east to west.

### The Guinea Worm Eradication Campaign

Several interventions have been used to end transmission of Guinea worm disease: health education (teaching people to filter their water through a finely woven cloth and not to enter water when they or their neighbors are infectious), safe drinking water from such sources as underground borehole wells, and vector control (using Abate).

The Guinea worm campaign, like other campaigns in the past, has illustrated the importance of political mobilization, including the mobilization of national leaders. For example, General Amadou Toumani Touré (a charismatic former head of state of the Republic of Mali), with the encouragement of President Carter in 1992, made the eradication of Guinea worm disease in Mali and in the nine other French-speaking countries in West Africa his personal mission.

The campaign faces a problem common to many other efforts to control infectious diseases in the industrialized and the developing world: underreporting. For example, in Ghana, as in Nigeria 10 years ago, and in many other countries, only three or four thousand cases of Guinea worm disease were officially reported; but the actual numbers were much higher. In 1989 when Ghana conducted a nationwide village-by-village search, almost 180,000 cases were found. Sudan began its eradication program late because of civil war. In 1996 and 1997, an apparent decline of cases in Sudan was due to less complete reporting because of increased fighting in 1997.

### The Campaign's Implications for Other Diseases

The Guinea worm campaign has demonstrated very graphically the possibility of village-based monthly reporting in Africa. In Ghana and Nigeria at the beginning of this program 10 years ago, such reporting did not exist. Now in those countries, more than 6,000 disease-endemic villages have volunteers who report to the national capital monthly.

The Guinea worm campaign has also demonstrated very clearly the efficacy of health education. In the beginning, many were skeptical because Guinea worm could not be combated with a vaccine, and eradication efforts had to rely on behavior change. However, behavior has changed. While we have been successful in helping to bring safe drinking water to many disease-endemic villages, the fastest and most effective intervention has been health education, which helped people understand where the parasite was coming from, how they were being infected, and the importance of using cloth filters to protect themselves and their families.

The campaign has underscored the potential of local volunteers. Many years ago in the Americas, village volunteers were used as part of malaria control efforts. The onchocerciasis control program in Africa is also using village volunteers successfully. The Guinea worm campaign has been another illustration of how volunteers can be used to diagnose, report, and provide, in this instance, on-the-spot treatment to neighbors for a specific infection. Those responsible for the campaign's success are often not members of the general health services.

With the help of the World Bank, the Guinea worm campaign demonstrates the importance of disease eradication to the national economy. The World Bank has estimated that the economic rate of return on the investment in Guinea worm eradication will be on the order of 29% per year once the disease is eradicated. That figure is based on a very conservative estimate of the average amount of time infected workers are unable to perform agricultural tasks.

The campaign has also created a group of trained health-care workers of a different generation from those involved in the smallpox eradication program. These workers have gone from beginning to end, from hearing the doubters and seeing the difficulty of initiating the campaign to tasting victory in their own countries. These workers can contribute to subsequent campaigns. Moreover, the concept of eradication, which was in disrepute only 5, 10 years ago, has been revived. Soon we will confirm that a nonviral disease for which vaccine is not available can be eradicated.

Like the smallpox eradication campaign, the Guinea worm campaign has illustrated very vividly in many different ways and at many different levels (from international to village level) the power of data. In the Guinea worm campaign, we have used surveillance data to promote health policy. One key lesson from the smallpox campaign we are deliberately applying in the Guinea worm campaign is to distill what needs to be done in terms of interventions to a handful, or almost a handful, of indexes (seven on an international level) to know what is most important and (as rapidly as possible) how well we are doing. That unleashes inordinate amounts of energy.

Finally, the Guinea worm eradication campaign will have illustrated again the power of demonstration. Eradication can happen because it has happened.

